# Knowledge and Attitude of Patients Attending Primary Health Care (PHC) or Family Medicine Clinics About Periodic Health Assessment

**DOI:** 10.7759/cureus.57616

**Published:** 2024-04-04

**Authors:** Mohammed Aldosari, Khalid M Alduraibi, Abdullaziz A Alsahly, Soliman A Alaraidh, Abdulrahman K Alsaleem, Mohammad S Almosa, Alwaleed Albarkani, Saleh Alhussaini

**Affiliations:** 1 Family Medicine, King Abdulaziz Medical City, Riyadh, SAU; 2 College of Medicine, King Saud Bin Abdulaziz University for Health Sciences, Riyadh, SAU

**Keywords:** riyadh, patient, family medicine clinics, attitude, knowledge, health assessment

## Abstract

Background

This study assesses the awareness and perceptions of periodic health assessments (PHA) among patients at primary health care (PHC) and family medicine clinics. Despite PHC's importance in preventive health, previous research indicates a significant gap in the public's knowledge and routine practice of PHA, potentially affected by various demographic factors.

Materials & methods

A cross-sectional approach was employed in Riyadh, Saudi Arabia, with data gathered via self-administered questionnaires from 382 participants. The survey focused on socio-demographic information, knowledge about PHA, and attitudes towards it. Statistical analysis explored the influence of demographic and clinical factors on individuals’ knowledge and attitudes.

Results

Findings showed that 300 (78.5%) participants had engaged in routine medical examinations, displaying substantial knowledge of PHA. Nevertheless, issues like healthcare accessibility and provider availability were identified as the major barriers, affecting 125 (32.7%) and 84 (22%) participants, respectively. The overall attitude towards PHA was positive, especially among individuals with chronic conditions, emphasizing its perceived benefits in health management.

Conclusion

The research underscores a generally positive attitude and fair knowledge level regarding PHA among the studied population, alongside significant barriers to participation. Targeted interventions that address these barriers and capitalize on the positive attitudes may enhance PHA uptake, promoting better health outcomes. This study contributes to the understanding of public engagement with PHA, offering insights for improving health promotion and disease prevention strategies.

## Introduction

Primary health care (PHC) has been periodically redefined since 1978 [1]. A simple definition was developed to facilitate the coordination of primary healthcare efforts across the globe. "Primary health care is a whole-of-society approach to health that aims at ensuring the highest possible level of health and well-being and their equitable distribution by focusing on people’s needs as early as possible along the continuum from health promotion and disease prevention to treatment, rehabilitation and palliative care, and as close as feasible to people’s everyday environment" [1]. Furthermore, primary healthcare is a comprehensive healthcare system. Therefore, it focuses on the person as a whole through the lifespan, rather than a specific disease or a life stage. One of the most fundamental principles of health care is to provide the highest standards of care to all people, without exception [2].

Healthcare prices have been on the rise. According to one study, healthcare expenditures in the United States reached $4.3 trillion in 2021 [3]. Primary health care is the most cost-efficient approach to enhance people’s physical health, mental health, and social well-being [1]. Moreover, it acts as a front door for detecting early signs of epidemics. It allows the healthcare system to respond early to different health service crisis demands. Primary healthcare practitioners are trained comprehensively, allowing them to detect the patient's healthcare needs in early disease stages. As such, patients can be referred and guided to other specialties through primary healthcare facilities or managed in the facility, depending on the health issue [4].

A study by Al-Etesh et al. (2020) in Al-Jouf Region, Saudi Arabia, showed results that concluded most of the Saudi adult participants in Al-Jouf Region (64%) lacked prior knowledge about the adult Periodic Health Evaluation (PHE) and 75% did not routinely practice it. The main reason for this was a lack of adult PHE informational sources among the community of the Al-Jouf Region in KSA [5].

Furthermore, a study by Ali et al. (2015) showed that only 22.5% of participants performed routine medical checkups, while the majority of them (77.5%) did not routinely perform checkups. In the study, they suggest that some factors such as age, gender and marital status may have an effect on the performance of routine checkups. Contrariwise, educational level, financial standing and occupational status appeared to have no effect on the performance of regular checkups. The majority of participants who practiced routine checkups did so out of personal conviction and belief in its benefits. And for those who do not practice routine checkups the most common reasons for avoiding routine checkups were not having enough time and a perception that medical check-ups were a long and boring process [6].

As we concluded previously, the goal of primary health care is to promote health and well-being at the highest level for all people and provide them with the needed treatment for any of their illnesses, either chronic care or acute, and transfer them as needed [1]. Moreover, it is the most cost-efficient and viable option of care available for all. It is also equipped with tools that could assist in patient care, and has competent health practitioners [1]. That is why, we aim to identify the level of awareness of the patients attending primary health care clinics about PHC and their thoughts in regards to all aspects of primary health assessment, its practitioners, and quality of care, which in turn will help improve the quality of PHC and the health care services provided for patients.

Objective of the study

The aim of this study is:

1- To assess the level of knowledge that patients have about periodic health assessment, including the benefits, recommended frequency, and the types of screening tests that are typically included.

The primary objectives of the study include:

1- Identifying any demographic or clinical factors that may be associated with differences in knowledge or attitudes towards periodic health assessment, such as age, gender, education level, or presence of chronic diseases.

2- To determine the attitudes of patients towards periodic health assessment, including their perceived importance, willingness to undergo screening tests, and any barriers or concerns they may have.

## Materials and methods

A cross-sectional study was conducted at primary health care and family medicine clinics in NGHA, Riyadh, Saudi Arabia. The co-investigators collected the data using a self-administered questionnaire. The study was conducted by reviewing questionnaire results from patients attending primary health care and family medicine clinics. A non-probability consecutive sampling technique was used by including all patients who meet the inclusion criteria.

The inclusion criteria encompass patients attending family medicine clinics or primary health care, including both male and female patients who are Saudi, whereas the exclusion criteria apply to patients not attending primary health care (PHC) or family medicine clinics and those who are non-Saudi.

The questionnaire underwent validation and reliability checks through assessments by public health experts. Subsequently, a pilot test involving a small varied sample from the target population was carried out. Feedback encompassing clarity, length, and comprehensiveness of the questionnaire was taken, and necessary adjustments were made. Patients were approached during their visit in general OPDs by one of the research members and it was a self-administered questionnaire. Patients were informed that their participation was voluntary, and their responses were kept confidential. The questionnaire was distributed to patients. The whole process took less than 10 minutes to complete. The questionnaires were translated into Arabic. The back translation was done by the experts to ensure the accuracy of the instruments. The description of the questionnaire is as follows:

The first part was socio-demographic: age, sex, education level, employment status, income, smoking, and chronic illnesses.

The second part items that measure knowledge about PHE, were adapted from another study “A Cross Section Survey Assessment Study on the Knowledge and Practice of Periodic Medical Checkup among the Saudi population” [7].

The third section which seeks to assess attitudes towards PHA was adapted from the same aforementioned study. This section gauges respondents' preferences regarding the mentioned factors. The questionnaire underwent revision and editing by a family medicine consultant.

A common grading method used for knowledge questions was as follows: 1 point was given to the correct option and 0 for the incorrect answer. After data collection, a participant who correctly answered more than 50% of the questions (4 or more points out of 6) was considered as having good knowledge about periodic health assessments.

Participants consented to use the data before involvement in the study. All data were kept safely and no identification data were asked, such as MRN, names, and ID (MRN was replaced with serial number). Subjects’ privacy and confidentiality were assured, no identifiers were collected, and all data were kept in a secure place within NGHA premises, both hard and soft copies. The access to research data was kept only between the group members.

Statistical analysis plan

SPSS software version 23.0 (IBM Corporation, Armonk, NY, USA) was used for statistical analysis. Mean and standard deviation were used for quantitative variables. Qualitative variables were presented using illustrative quotes. The chi-square test and Fisher's exact test were used to compare the qualitative variables. The significant p-value was less than 0.05.

## Results

About 382 participants completed the questionnaires. Out of them, 72.8% were males and 27.2% were females. Most of the participants (66.8%) were married and 29.1% were singles during the period of the study. Regarding their level of education, 51% of the participants were bachelor’s degree holders, 29.8% had secondary school education, 8.1% had master's degree, 5.2% had intermediate school education, 2.4% were elementary educated, 2.1% had doctorate degree, and only 1.3% were uneducated. The study included 49 students (12.8%), 163 employed individuals (42.7%), 67 unemployed individuals (17.5%) and 103 retired individuals (27%). Most of the participants have an income of 10,000 to 20,000 SR (38%). About 25.4% of the participants were smokers and 45% had chronic diseases (Table 1).

**Table 1:** Socio-demographic characteristics of the study participants (n=382) SAR: Saudi Arabian Riyal

Variable	Categories	Frequency	Percent
Gender	Male	278	72.8
	Female	104	27.2
Marital status	Single	111	29.1
	Married	255	66.8
	Divorced	8	2.1
	Widower	8	2.1
Educational level	Uneducated	5	1.3
	Elementary	9	2.4
	Intermediate	20	5.2
	Secondary	114	29.8
	University	195	51
	Master's	31	8.1
	Doctorate	8	2.1
Employment status	Student	49	12.8
	Employee	163	42.7
	Unemployed	67	17.5
	Retired	103	27
Financial Income to the family (SAR)	Less than 10,000	107	28
	10,000 - 20,000	145	38
	20,000 - 30,000	97	25.4
	> 30,000	33	8.6
Smoking	Yes	97	25.4
	No	285	74.6
Chronic illnesses	Yes	172	45
	No	210	55

**Figure 1:**
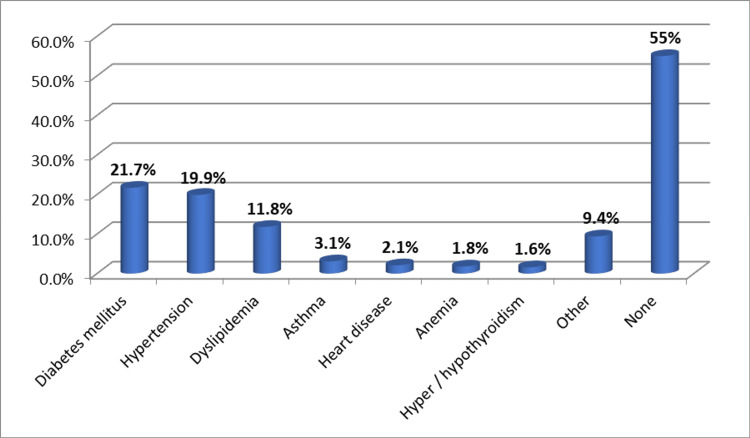
Frequency of chronic diseases among the study participants

Chronic illnesses among the participants included: diabetes mellitus (21.7%), hypertension (19.9%), dyslipidemia (11.8%), asthma (3.1%), cardiac disease (2.1%), anemia (1.8%), hyperthyroidism and hypothyroidism (1.6%) and other diseases (9.4%) while 55% of the participants did not have any chronic illnesses (Figure 1).

Regarding the participant's knowledge about periodic health assessment, 78.5% of them had a routine medical examination and a lower percentage of them (53.7%) had a regular medical checkup. Regarding the frequency of medical checkups, most participants had a checkup every six months (35.6%). Blood pressure test was the most requested test in periodic medical checkups (45.3%) followed by diabetes tests (41.9%) then visual and dental examinations (14.4% and 13.9%, respectively). The most reported causes for poor implementation of periodic medical examinations were distance from the health center (32.7%) and lack of doctors (22%). More than two-thirds of the participants (77%) observed improvement during medical checkups and the vast majority of them (94.8%) thought that periodic medical checkups help to discover early problems while more than half (57.1%) of the participants said that there were no complications because of lack of periodic medical checkups (Table 2).

**Table 2:** Knowledge of periodic health assessment N: frequency, %: percentage

Variable	Categories	N (%)
Have you ever had a routine medical examination?	Yes	300 (78.5)
	No	82 (21.5)
Do you have regular medical checkups?	Yes	205 (53.7)
	No	177 (46.3)
Frequency of medical checkup	Every 6 months	136 (35.6)
	Every 1 year	97 (25.4)
	Every 2 years	17 (4.5)
	I don’t know	132 (34.6)
Types of periodic medical checkups known	Blood pressure tests	173 (45.3)
	Diabetes tests	160 (41.9)
	Visual examination	55 (14.4)
	Dental examination	53 (13.9)
	All of the above	178 (46.6)
Causes of poor implementation of periodic medical checkups	Lack of Doctors	84 (22)
	Distance from the health center	125 (32.7)
	Lack of time	19 (5)
	Lack of Money	41 (10.7)
	Unawareness	19 (5)
	Unavailability or difficulty booking appointments	10 (2.6)
	Poor scheduling	10 (2.6)
	Length of appointments	6 (1.6)
	Other	36 (9.4)
Any improvement during medical checkup	Yes	294 (77)
	No	88 (23)
Any complications because of lack of periodic medical checkups	Yes	164 (42.9)
	No	218 (57.1)
Is it helpful to discover early problem?	Yes	362 (94.8)
	No	20 (5.2)

Regarding the participant's attitude toward periodic health assessment, most of them (58.4%) strongly agreed that periodic health assessment improves the prognosis of chronic illnesses. About 25.9% of the participants strongly agreed that regular visits to the doctor are difficult and a similar percentage of them (25.4%) agreed a little with that. Regarding periodic health assessments only for specific groups of patients, 26.4% strongly agreed with that and 21.5% totally disagreed that they don't go to check-ups regularly because they are healthy. Periodic health assessment reduces the risk of complications and helps to detect some diseases early, this was strongly agreed by 58.4% and 61.5% of the participants, respectively. A lower percentage of the participants strongly agreed that periodic health assessments protect against malignancy and help to protect against chronic illnesses (38% and 43.5%, respectively). About one-third of the participants (35.9%) visit their doctors annually and more than half (58.1%) of the participants strongly agreed that it's important to visit their doctor annually (Table 3).

**Table 3:** Attitude toward periodic health assessment N: frequency, %: percentage

Variable	Strongly agree, N (%)	Totally agree, N (%)	Agree a little, N (%)	Disagree a little, N (%)	Totally disagree, N (%)	Strongly disagree, N (%)
Periodic health assessment improves the prognosis of chronic illnesses	223 (58.4)	132 (34.6)	21 (5.5)	1 (0.3)	3 (0.8)	2 (0.5)
I find visiting the doctor regularly is difficult	99 (25.9)	85 (22.3)	97 (25.4)	28 (7.3)	35 (9.2)	38 (9.9)
Periodic health assessment only for specific groups of patients	101 (26.4)	82 (21.5)	68 (17.8)	47 (12.3)	28 (7.3)	56 (14.7)
I don't go to check-up regularly because I'm healthy	52 (13.6)	52 (13.6)	68 (17.8)	55 (14.4)	82 (21.5)	73 (19.1)
Periodic health assessment reduces the risk of complications	223 (58.4)	128 (33.5)	25 (6.5)	3 (0.8)	1 (0.3)	2 (0.5)
Periodic health assessment helps to detect some diseases early	235 (61.5)	122 (31.9)	18 (4.7)	1 (0.3)	4 (1)	2 (0.5)
Periodic health assessment protects against malignancy	145 (38)	103 (27)	69 (18.1)	23 (6)	22 (5.8)	20 (5.2)
Periodic health assessment helps to protect against chronic illnesses	166 (43.5)	117 (30.6)	61 (16)	16 (4.2)	11 (2.9)	11 (2.9)
I visit my doctor yearly	137 (35.9)	91 (23.8)	64 (16.8)	32 (8.4)	23 (6)	35 (9.2)
It's important to visit my doctor yearly	222 (58.1)	109 (28.5)	38 (9.9)	3 (0.8)	4 (1)	6 (1.6)

Out of a total score of 6, the mean knowledge score of the participants was 4.1 ± 1.6, and 259 (67.8%) participants were considered as having good knowledge about periodic health assessment (correctly answered more than 50% of the knowledge questions) while 123 (32.2%) had poor knowledge. The average attitude score of the participants was 7.6 ± 1.3 out of 10. A total of 360 participants (94.2%) were considered to have a positive attitude toward periodic health assessment while 22 (5.8%) had a negative attitude.

Statistical tests were used to explore the relationship between knowledge and attitude toward periodic health assessment and different socio-demographic factors. There was a significant association between marital status (P < 0.001), employment status (P < 0.001), financial income (P = 0.030) and chronic diseases (P < 0.001) in relation to the knowledge about periodic health assessment. Divorced, elementary educated, retired participants, participants with 10,000 to 20,000 SAR income and participants with a history of chronic disease showed a higher level of knowledge compared to others. Chronic illnesses were the only factor that was significantly associated with attitude toward periodic health assessment (P = 0.030); participants who have chronic disease had higher levels of positive attitudes compared to those without a history of chronic diseases. Other socio-demographic factors did not reach the statistical significance level (p < 0.05) (Table 4).

**Table 4:** Socio-demographic factors associated with knowledge and attitude toward periodic health assessment N: frequency, %: percentage, F: p-value calculated using Fisher's exact test, other p-values calculated using Chi-square test. * Significant p-value < 0.05.

Variable	Level of knowledge		P-value	Attitude		P-value
	Good N (%)	Poor N (%)		Positive N (%)	Negative N (%)	
Gender						
Male	182 (65.5)	96 (34.5)	0.111	261 (93.9)	17 (6.1)	0.625
Female	77 (74)	27 (26)		99 (95.2)	5 (4.8)	
Marital status						
Single	45 (40.5)	66 (59.5)	< 0.001^F^*	100 (90.1)	11 (9.9)	0.128^F^
Married	199 (78)	56 (22)		245 (96.1)	10 (3.9)	
Divorced	8 (100)	0 (0)		7 (87.5)	1 (12.5)	
Widower	7 (87.5)	1 (12.5)		8 (100)	0 (0)	
Educational level						
Uneducated	2 (40)	3 (60)	0.095^F^	5 (100)	0 (0)	0.178^F^
Elementary	7 (77.8)	2 (22.2)		7 (77.8)	2 (22.2)	
Intermediate	15 (75)	5 (25)		19 (95)	1 (5)	
Secondary	87 (76.3)	27 (23.7)		111 (97.4)	3 (2.6)	
University	120 (61.5)	75 (38.5)		181 (92.8)	14 (7.2)	
Master's	23 (74.2)	8 (25.8)		30 (96.8)	1 (3.2)	
Doctorate	5 (62.5)	3 (37.5)		7 (87.5)	1 (12.5)	
Employment status						
Student	16 (32.7)	33 (67.3)	< 0.001*	45 (91.8)	4 (8.2)	0.204^F^
Employee	104 (63.8)	59 (36.2)		153 (93.9)	10 (6.1)	
Unemployed	45 (67.2)	22 (32.8)		61 (91)	6 (9)	
Retired	94 (91.3)	9 (8.7)		101 (98.1)	2 91.9)	
Financial Income (SAR)						
Less than 10,000	65 (60.7)	42 (39.3)	0.030*	101 (94.4)	8 (5.6)	0.833
10,000 - 20,000	99 (68.3)	46 (31.7)		135 (93.1)	10 (6.9)	
20,000 - 30,000	76 (78.4)	21 (21.6)		92 (94.8)	5 (5.2)	
> 30,000	19 (57.6)	14 (42.4)		32 (97)	1 (3)	
Chronic illnesses						
Yes	149 (86.6)	23 (13.4)	< 0.001*	167 (97.1)	5 (2.9)	0.030*
No	110 (52.4)	100 (47.6)		193 (91.9)	17 (8.1)	

## Discussion

This study provides a comprehensive overview of the socio-demographic characteristics, knowledge, and attitudes towards periodic health assessments among a sample of 382 participants. In this study, we observed a predominance of male participants (72.8%) compared to females (27.2%). This gender distribution may reflect specific cultural, social, or occupational contexts that influence participation in health-related studies. Previous research has often indicated varying health-seeking behaviors among genders, with women generally more likely to engage in preventive health measures than men [6]. Educational level played a crucial role in the knowledge about periodic health assessments, with bachelor's degree holders constituting the majority (51%). This is coherent with findings from Zhang et al. (2021), as these authors discovered that higher educational levels are linked to the better health literacy [8]. Nevertheless, we can compare our results with studies that showed an association between low educational levels and low PHE levels. Notably, the findings from this study revealed a significant association between knowledge level and employment status, mostly highlighting retired individuals as having higher knowledge which could be attributed to the more frequent interaction they have with healthcare systems and possibly more time dedicated to the health issues [4]. Financial income was another determinant of knowledge where individuals with an income of 10,000 to 20,000 SAR had better knowledge. This implies that the economic stability enables access to health information and services [9].

The prevalence of chronic illnesses in our study (45%) and its strong association with the positive attitude towards periodic health assessments indicate the necessity of such evaluations for individuals with chronic conditions. This coincides with the study by Chien et al. (2020), who indicated that having chronic illnesses influences individuals to participate regularly in health screenings so as to effectively manage their health conditions [10].

Regarding knowledge about periodic health assessment, 78.5% of the respondents had engaged in routine medical screening which shows that most people are aware of the importance of health screening. This high engagement rate implies a proactive approach to health management within the community, hence the increasing interest in preventive health care in terms of advocacy [11]. Literature has consistently demonstrated that chronic conditions can be effectively managed through early detection and regular health checkups which will lead to better health outcomes and reduced healthcare costs. For example, Maqbul et al. (2021) highlight the importance of preventive services in the management of chronic diseases. This function is underlined by the high levels of engagement in blood pressure and diabetes tests by the study participants [12]. Nevertheless, the gap between awareness and utilization of different types of checkups, which include the visual and the dental examination, poses a concern similar to the inadequacies reflected in public health education studies which stress that oral health is often ignored to be a factor in quality of life [12,13].

The identification of barriers, such as distance from health centers and lack of doctors, reflects a common challenge in global health literature. One of the primary challenges to attaining universal health coverage, especially in low- and middle-income countries, is the availability of healthcare services, which the World Health Organization (WHO) has long acknowledged. The barriers mentioned in this study are similar to those presented in the literature, pointing to the need for healthcare systems to undergo changes that would make them more available to serve the population [1]. A framework for comprehending and resolving these barriers has been established through studies investigating the aspects of access, which include acceptability, affordability, accommodation, accessibility, and availability [6].

The positive attitudes towards periodic health assessments based on the strong agreement on their benefits for the prognosis of chronic illnesses and detecting diseases early are consistent with the preventive care model commonly discussed in healthcare. Studies such as those by Vuong (2016) on the stages of change model suggest that positive attitudes towards health behaviors are crucial in the transition from contemplation to action and maintenance of health-promoting behaviors [14]. However, the acknowledgment of difficulties in visiting the doctor regularly points to an area where practical solutions are needed to bridge the gap between positive attitudes and actual health-seeking behavior. This dichotomy between attitude and action is a well-documented phenomenon in health psychology, known as the intention-behavior gap [5,15].

The significant association between marital, educational, and employment status with knowledge about periodic health assessments suggests that socio-economic factors play a critical role in health literacy and engagement. In a study conducted by Ming et al. (2017), married men are more inclined to seek medical care compared to unmarried men, mostly because of the influence exerted by their female partners. This is particularly evident in situations involving regular health check-ups [16]. Particularly, divorced, elementary educated, and retired participants showing a higher level of knowledge could reflect targeted health education or personal experiences navigating health challenges. Interestingly, chronic illnesses were significantly associated with a positive attitude towards periodic health assessments. This finding suggests that personal health conditions may motivate individuals to adopt a more proactive approach to health maintenance [17].

Strengths and limitations

Our study has some notable strengths. By focusing on periodic health assessments, the study addresses a crucial component of preventive healthcare, offering insights that can inform policy and practice in health promotion and disease prevention. Our study also identifies specific barriers to engaging in periodic health assessments, such as distance from health centers and lack of doctors, providing valuable information for healthcare planners and policymakers to address these issues. On the other hand, there are a few limitations to this research. The study's cross-sectional design makes it difficult to draw any firm conclusions about the relationship between demographic variables and participants' awareness of, or comfort with, the need of routine health screenings. Secondly, answering questions on PHA use and knowledge was dependent on participants' self-reported data. Response bias might affect the findings if participants provide responses that are socially acceptable or if they don't remember their own attitudes or behavior correctly. Because the research was carried out in NGHA, Riyadh, Saudi Arabia, a particular geographic and cultural setting, it's possible that the results cannot be generalized to other populations or areas with different healthcare systems or cultural perspectives on health.

## Conclusions

This study has identified a generally positive attitude and a fair level of knowledge about periodic health assessments among the participants. Despite the positive attitudes, the study identifies barriers to participation in periodic health assessments, such as accessibility issues and a lack of healthcare providers. The association between socio-demographic factors and the level of knowledge and attitudes towards health assessments suggests that targeted interventions could enhance public engagement in preventive health measures. Addressing the identified barriers and leveraging the insights about socio-demographic associations with knowledge and attitudes can guide the development of strategies to improve the uptake of periodic health assessments. Moreover, by focusing on preventive healthcare, policymakers and healthcare providers can work towards achieving higher standards of health and well-being for the population.
